# A modified PATH algorithm rapidly generates transition states comparable to those found by other well established algorithms

**DOI:** 10.1063/1.4941599

**Published:** 2016-02-24

**Authors:** Srinivas Niranj Chandrasekaran, Jhuma Das, Nikolay V. Dokholyan, Charles W. Carter

**Affiliations:** Department of Biochemistry and Biophysics, The University of North Carolina at Chapel Hill, Chapel Hill, North Carolina 27599-7260, USA

## Abstract

PATH rapidly computes a path and a transition state between crystal structures by minimizing the Onsager-Machlup action. It requires input parameters whose range of values can generate different transition-state structures that cannot be uniquely compared with those generated by other methods. We outline modifications to estimate these input parameters to circumvent these difficulties and validate the PATH transition states by showing consistency between transition-states derived by different algorithms for unrelated protein systems. Although functional protein conformational change trajectories are to a degree stochastic, they nonetheless pass through a well-defined transition state whose detailed structural properties can rapidly be identified using PATH.

## INTRODUCTION

Computational treatments of protein conformational changes tend to focus on the trajectories themselves, despite the fact that it is the transition state structures that contain information about the barriers that impose multi-state behavior. Enzymatic reactions often take place in multiple steps. Slower, protein conformational changes can be rate-limiting even if the catalyzed chemical reaction is rapid.^39^ Such conformational transitions act as molecular timers to help regulate amplitude and duration of cellular processes.^28^ High-energy configurations, or conformational transition states, therefore impose discrete multi-state behavior on proteins,^21^ significantly enhancing function by creating the capacity for a protein to transmit time and ligand-dependent information and/or mechanical motion necessary for signaling and other free-energy transduction processes. Structures of conformational transition states should therefore reveal valuable information about the energy barriers that separate one equilibrium structure from another. Understanding conformational transitions, however, requires characterizing the conformational transition states, an approach that is akin to understanding chemical reactions by characterizing their chemical transition states.

The structural reaction profile of *Geobacillus stearothermophilus* tryptophanyl-tRNA synthetase (TrpRS) involves three conformationally distinct states^6^ that impose rate-limiting conformational changes.^40,41^ Attempts to understand those rate-limiting conformations have led to two studies in which we showed that the PATH algorithm^18^ suggested previously unsuspected consistency with Molecular Dynamics (MD) simulations^23^ and steady-state kinetics measurements of TrpRS catalysis^41^ and TrpRS structural reaction path. The present work was therefore undertaken in order to validate those conclusions, and in particular to assess the generality with which PATH identifies appropriate conformational transition-state structures. We begin by describing several of the algorithms now in use to simulate trajectories for conformational changes—as distinct from protein folding reactions. Then, we outline the PATH algorithm with particular emphasis on the conceptual difficulties it poses and an approach that circumvents most of these difficulties, enhancing its general utility. We conclude by describing three new results that furnish complementary validation of the transition state structures identified by PATH.

In summary:

• We confirm and amplify observations summarized by Pinski and Stuart^33^ that minimizing the Onsager-Machlup (OM) functional requires optimized estimates of both the time taken for the transition and the energy difference between initial and final states, and that as such paths can involve non-physical features, they must be treated with caution, and hence validated by other types of information, which here include comparing different algorithms and molecular systems.
• These difficulties notwithstanding, distinct algorithms including PATH^18^ and ANMPathway^8^ produce quite similar transition-state structures to that generated using the temperature-dependent string method,^31^ which can be considered a “gold standard.”
• The transition state structure obtained by modifying the PATH algorithm to eliminate non-physical invariant portions of the trajectory coincides closely with the saddle point in the free energy surface simulated using Discrete Molecular Dynamics (DMD).^34,42^
• Cooperative repacking of aromatic side-chains is a common feature of transition state structures for domain rearrangements in three unrelated protein systems.

### Computational characterization of complex protein configurational landscapes

Considerable effort has been devoted to identifying structural features of the highest energy ensembles during protein folding.^7,10,12,13,30,34,36^ A general conclusion of that work is that multiple pathways can lead to the folded structures of many proteins, via an ensemble of related structures. We focus here on a much more restricted ensemble that occurs during conformational changes between distinct stable, folded structures formed as the result of ligand binding (i.e., allostery) and/or catalysis. The existence of such high-energy structures was suggested by the observation that removing ligands from MD simulations of two, quite similar structures representing the TrpRS Pre-transition state (Pre-TS) complex, caused them to relax rapidly, one toward Products, the other toward the Open ground state.^20,21^

The transient lifetimes of conformational transition states prevent access by traditional experimental approaches to their structures. Computational approaches, like MD simulations, do allow these states to be probed. Though successful for small proteins, conformational changes in large proteins occur on time scales that are several orders of magnitude larger and require intensive computational resources.

Two algorithms, among others, can increase the efficiency of searching the complex configuration spaces of large proteins. Replica exchange sampling^37^ furnishes a comprehensive mapping of the conformational free energy landscape.^42^ The string method^15,31^ furnishes an analytical algorithm for mapping the most probable path through such landscapes.

The replica exchange algorithm efficiently searches the configuration space of proteins by overcoming the sampling problem that affects single temperature simulations, which is that, at low temperatures the structures do not have enough energy to overcome conformational barriers and at high temperatures, the structures are unfolded and are far from the equilibrium states. In replica exchange simulations, multiple replicas of the starting structure are simulated at different temperatures and at defined time intervals, structures at different temperatures are exchanged. By doing this, replica exchange simulations allow systems to explore structures at different temperatures, thus sampling the conformational landscape, quickly and efficiently.

The string method computes a most probable trajectory through the conformational free energy surface using intervals between nodes defined in terms of “collective variables” along the path. It describes the transition pathway as the curve that connects successive metastable states so as to maintain a tangential projection of the curvature of the collective variables with respect to Cartesian space onto the free energy surface defined by the collective variables. This procedure can be seen as an application of the chain rule. Using collective variables reduces the number of degrees of freedom over which MD simulations are required.

The progress between successive states is monitored in the String method with the help of a reaction coordinate called the committor function, which is the fraction of molecules that complete the trajectory from each node. The transition state along a trajectory between the two equilibrium states is achieved when the committor function reaches a value of 0.5. The all-atom Chemistry at HARvard Molecular Mechanics (CHARMM) potential and the analytical formulation of the gradient mean that the string method can be considered to be the gold standard in the field. In spite of the success of the string method, it is nevertheless resource intensive.

Many functional conformational changes are distinct from protein folding reactions in that they entail primarily large amplitude motions that are independent of individual covalent bond vibration. Often, these conformational changes are rigid-body motions that can be replicated by the superposition of a few large amplitude normal modes. Numerous algorithms have been introduced to exploit Elastic Network Models (ENM)^2^ in the computation of conformational change trajectories,^26,43^ based either on minimizing the path integral of a free energy functional corresponding to the action or “resistance” along the path^26^ or on incremental searches from the initial and final states along the direction of a distance vector connecting the two states.^43^ The former approach has the appeal of producing a differentiable curve through the centers of a smooth tube in pathspace containing the most probable paths.^14,33^ Another related algorithm is ANMPathway,^8^ which uses an Anisotropic Network Model (ANM)^1^ to describe the potential energy wells of the two equilibrium states. This method requires two types of energy minimization steps that are performed, one within the cusp hypersurface at the transition state, and the other in the energy wells of the two states.

Curiously, despite the relative importance of conformational transition states, few, if any of the computational studies on conformational changes to date have focused on the transition state structures. We argue that in many ways transition-state structures, not the exact path, may be what are most important about conformational transitions. In this paper, we therefore investigate further the possibility that these simplified potentials may furnish a sufficient basis set to identify valid transition state structures for such motions. Thus, whereas most treatments focus on the trajectories; we focus here on the transition states themselves because they contain information about the barriers that impose multi-state behavior on proteins.

### PATH rapidly computes the most likely path and transition state

PATH (formerly MinActionPath^18^) is an algorithm that rapidly computes conformational transition states and the associated trajectories by minimizing the OM functional. The probability of finding a stochastic system at a given position and time is given by the Fokker-Planck equation. The OM functional is derived from the solution to the Fokker-Planck equation,^29^ such that its minimization by a variational computation, implemented using the Euler-Lagrange equations, furnishes equations of motion describing the most probable path.

PATH defines the structures of equilibrium states using a linearized ANM potential. This approximation of the complex potential energy landscape works because most protein conformational changes are small displacements from the equilibrium states. PATH uses either all atom or more limited ANM models to identify the transition state. Then, it computes paths to and from that transition state using the OM equations of motion. It also computes the time to the transition state, which is formally the reciprocal of a rate. The ratio of forward to reverse rates potentially can be used to estimate the equilibrium constant, and hence the free energy difference associated with the conformational change. This algorithmic difference means that potentially useful kinetic and thermodynamic information might be obtained from PATH simulations. We deal only tangentially with thermodynamic and kinetic aspects in this report, in which we focus on structural characterization of transition states.

The main drawback with the current implementation of PATH^18^ is that several input parameters must be known before simulations can be set up. These input parameters include the force constants for the ANM descriptions of, and the free energy difference between, the initial and final states, as well as the total time allowed for the transition. This dependence of the PATH output transition-state structures on these input parameters limits comparisons with other simulation methods and experiments. In this paper, we modify the PATH algorithm and outline a method for choosing suitable values of these input parameters, thereby making PATH a more effective simulation algorithm for studying protein conformational changes.

### Minimum action pathways depend on input parameters

**FIG. 1.**
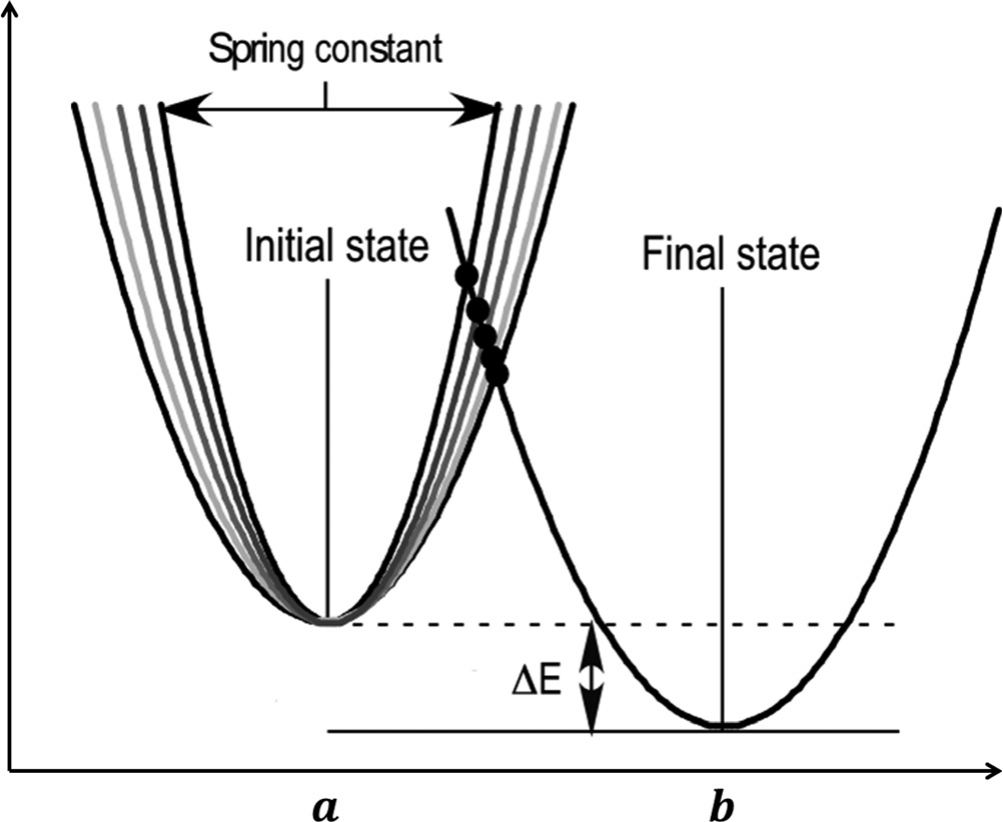
The two states of the protein as represented by the two wells. The width of the well is given by the magnitude of the force constant, larger the magnitude, narrower the well and vice versa. The difference in energy between the two minima is given by Δ*E*. In the current representation, the abscissa is the euclidean distance between the two minima. But in a different representation, the abscissa can also be the time axis with 0 at the first minimum and *t_f_* at the second minimum.

PATH models the two equilibrium structures, between which the path has to be computed, as harmonic potential wells and the point of intersection of the two wells as the transition state. The shapes of the harmonic wells are defined by force constants *k_l_* and *k_r_* for the left and the right potential wells, respectively (Fig. 1).

The two structures are input crystal structures, *a* and *b*, and the force constants are calculated from the Hessian matrix as described in Appendix B. At the point of intersection, which is the transition state $\bar{x}$, the two wells have the same energy *U*^‡^. If we consider the total time taken to make the full transition to be *t_f_*, then the time taken to reach the transition state from the initial state, $\bar{t}$, is a fraction of the total time and it uniquely identifies each minimum action path at that *t_f_*.

**FIG. 2.**
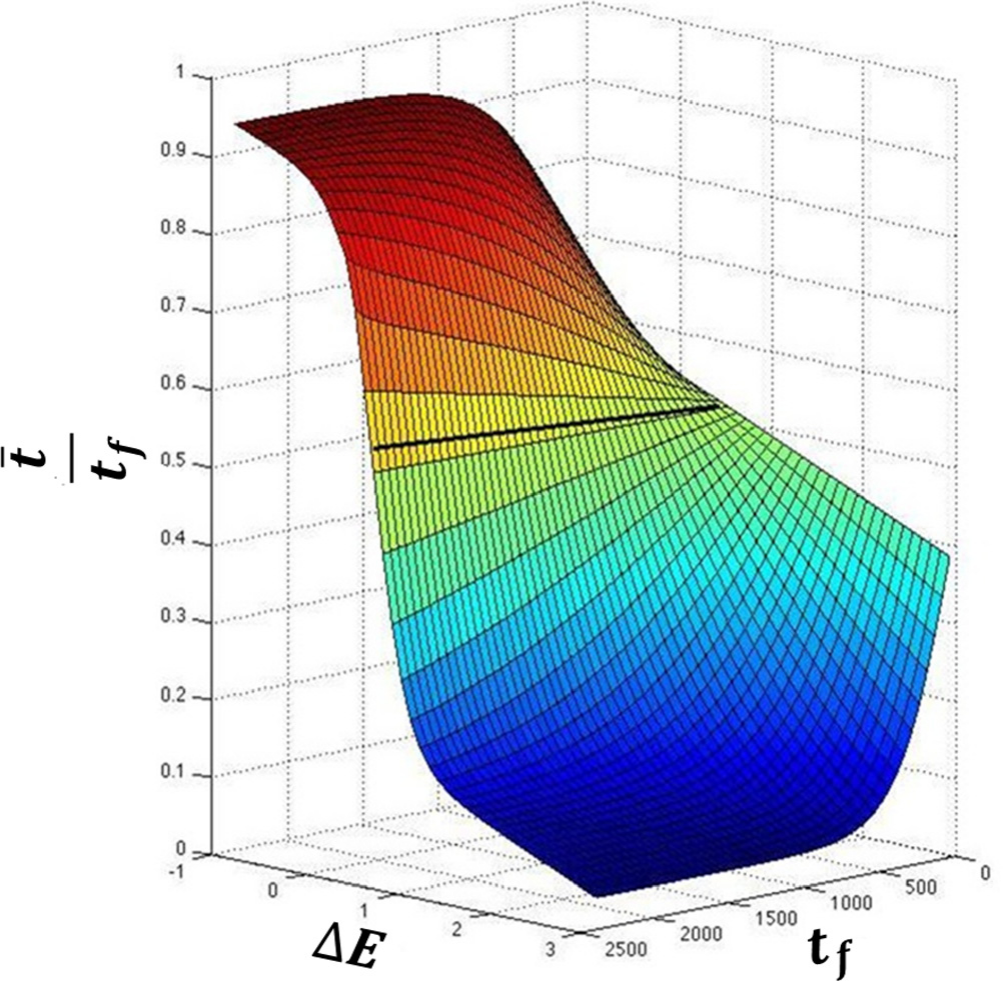
From Fig. 1, it can be seen that the path must depend on both *t_f_* and Δ*E*. Since $\bar{t}$, at each value of *t_f_*, uniquely identifies a path as a function of Δ*E*, it gives rise to the convergence surface shown in this figure. The surface was fitted to simulations of the catalytic step of TrpRS (*R*^2^ = 0.99). The surface shows a sigmoidal dependence of $\bar{t}$ on both Δ*E* and *t_f_*. Since only positive values of *t_f_* are used in the simulations, only the lower half of the sigmoid is seen along the *t_f_* axis and it can be fitted approximately to a rectangular hyperbola. This surface retains its shape for the diatomic system in one dimension.

From Fig. 1, it can be seen that if either force constant, *k_l_* or *k_r_*, the relative energy difference between the two wells, (Δ*E*), or *t_f_* are changed, then the minimum action path that the system will take would be different. This means that for different values of Δ*E* and *t_f_*, and as noted previously,^33^ there are different minimum action paths between the given equilibrium states, each defined by a different $\bar{t}$. As previously mentioned, since $\bar{t}$ uniquely identifies each path, when plotted against different values of Δ*E* and *t_f_*, it gives rise to the surface that we call the convergence surface (Fig. 2).

This surface represents all the possible minimum action trajectories between a given pair of structures and it is different for different pairs of structures. The bi-sigmoidal functional form of this surface is discussed in Appendix C. This surface also means that multiple, locally minimum action paths are possible for the same pair of structures. Appropriate values of both Δ*E* and *t_f_* must therefore be chosen to identify a single minimum action path and transition state that is closest to what is observed in nature.

As mentioned earlier, the force constants are calculated from the Hessian matrix, which is built using a scale constant that is obtained by fitting crystallographic B values to the mean-square fluctuations of atoms in the structure.^2^ Hence, their accuracy depends strongly on the resolution of the X-ray data. This restriction appears to limit the application of PATH to high resolution crystal structures. Alternately, force constants can, in principle, be determined iteratively by perturbative methods. Parameter estimation can thus require tens of simulations, compromising on the relative speed of PATH simulations. An alternate method to calculate the force constants must be used to elevate the applicability of PATH to that of a general method for studying protein conformational changes.

In the following, we describe these parameters in greater detail, in the context of PATH computations and the convergence surface, and then outline a general strategy for estimating appropriate values of these parameters.

## THEORY OF PATH

A significant advantage of the string method^31,38^ is that it calculates the Minimum Free Energy Path (MFEP) between two equilibrium states. The MFEP must be contrasted with the Minimum Energy Path (MEP) that minimizes the Freidlin-Wentzell action,^27^ which is the low temperature homolog of the Onsager-Machlup action. Thus, significant parallels emerge between MEP trajectories that minimize the latter action (previously termed “resistance”^3^) and MFEP that minimize the Onsager-Machlup action. The essential difference between the two approaches is that entropic changes play no role at zero temperature. Pinski and Stuart^33^ showed that the effects of temperature are of little significance, provided there was no energy difference between initial and final states, but that significant energy differences between states introduced comparable changes in the transition states obtained at different temperatures when minimizing the Freidlin-Wentzell action functional. The relevance of free-energy differences between initial and final conformational states emphasizes the value of minimizing the Onsager-Machlup action functional as a useful approximation to the computationally intensive string method. PATH^18^ implements such an algorithm.

PATH simulates the dynamics of a protein molecule by computing the solution to the equation of motion derived from the minimization of the Onsager-Machlup functional, using the Euler-Lagrange equations [Appendix A]. For studying conformational changes between two different equilibrium structures, PATH represents the two structures using a double well potential well (a linearized ANM) and uses a separate equation of motion for the dynamics within each potential well.

**FIG. 3.**
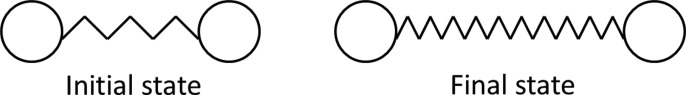
The diatomic system can be represented by the ball and spring model. In the two states, the distance between the two atoms is different, and also the strength of the spring (force constant) is also different. The diatomic system also affords an analytical functional form for the bi-sigmoidal dependence of the convergence surface on Δ*E* and *t_f_*.

In the case of a simple diatomic system in one dimension (Fig. 3), the Onsager-Machlup equations of motion are written as

$$x_{l}(t)=\{\begin{array}{cc} a+\left(\bar{x}-a\right)\left(\frac{\sinh\left(k_{l}t\right)}{\sinh\left(k_{l}\bar{t}\right)}\right) & when \end{array}\;t\leq \bar{t},$$ (1)

$$x_{r}(t)=\{\begin{array}{cc} b+\left(b-\bar{x}\right)\left(\frac{\sinh\left(k_{r}\left(t-t_{f}\right)\right)}{\sinh\left(k_{r}\left(t_{f}-\bar{t}\right)\right)}\right) & when \end{array}\;t\geq \bar{t},$$ (2)

where $\bar{t}$ is the time taken to reach the transition state, *t_f_* is the total time for transition, $\bar{x}$ is the transition state, *a* and *b* are the initial and the final states, respectively, *k_l_* and *k_r_* are the force constants for the initial and the final states, respectively.

For a smooth transition from one well to the other, the paths have to satisfy boundary conditions based on position and velocity. We express these conditions mathematically in the following way:

$$\begin{array}{c} x_{l}\left(t\to \bar{t}\right)=x_{r}(\bar{t}\leftarrow t_{f}),\\ {\dot{x}}_{l}\left(t\to \bar{t}\right)={\dot{x}}_{r}\left(\bar{t}\leftarrow t_{f}\right). \end{array}$$ (3)

Also $x_{l}\left(0\right)=a,\;x_{r}\left(t_{f}\right)=b$ and $x\left(\bar{t}\right)=\bar{x}$, where *x_l_* and *x_r_* are the trajectories in the left and right well, respectively.

For multiatom 3D system, the interactions between the atoms are more complex, and in the case of a linearized ANM the interaction matrix is a hessian matrix [Appendix B]. Then, the equations of motion can be written as

$$x_{l}\left(t\right)=V\left[\left(\begin{array}{cc} \frac{t}{\bar{t}} & 0\\ 0 & \frac{\sinh\left(\lambda_{l}^{i}t\right)}{\sinh\left(\lambda_{l}^{i}\bar{t}\right)} \end{array}\right)\bar{\psi }\right]+a,$$ (4)

$$x_{r}\left(t\right)=W\left[\left(\begin{array}{cc} \frac{t_{f}-t}{t_{f}-\bar{t}} & 0\\ 0 & -\frac{\sinh\left(\lambda_{r}^{i}\left(t-t_{f}\right)\right)}{\sinh\left(\lambda_{r}^{i}\left(t_{f}-\bar{t}\right)\right)} \end{array}\right)\bar{\phi }\right]+b,$$ (5)

where $\bar{\psi }=V^{T}\left(\bar{x}-a\right),\;\bar{\phi }=W^{T}\left(\bar{x}-b\right)$. *V* and *W* are the eigenvectors of the Hessian matrices of the initial and final wells, and $\lambda_{l}^{i}$ and $\lambda_{r}^{i}$ are their eigenvalues. The eigenvalues replace the force constants in the trajectory equations because by diagonalizing the Hessian matrix, we generate 3N normal modes whose individual motion depends on the rate at which the structure changes, which is given by the eigenvalues. The final trajectory is generated by a linear combination of the normal modes.

To solve for the transition state, we apply velocity continuity

$$V\left[\underset{L}{\underset{︸}{\left(\begin{array}{cc} \frac{1}{\bar{t}} & 0\\ 0 & \frac{\lambda_{l}^{i}\cosh\left(\lambda_{l}^{i}\bar{t}\right)}{\sinh\left(\lambda_{l}^{i}\bar{t}\right)} \end{array}\right)}}\bar{\psi }\right]=W\left[\underset{R}{\underset{︸}{\left(\begin{array}{cc} \frac{1}{\bar{t}-t_{f}} & 0\\ 0 & \frac{\lambda_{r}^{i}\cosh\left(\lambda_{r}^{i}\left(\bar{t}-t_{f}\right)\right)}{\sinh\left(\lambda_{r}^{i}\left(\bar{t}-t_{f}\right)\right)} \end{array}\right)}}\bar{\phi }\right],$$ (6)

which can be rewritten, to compute the transition state, as

$$\bar{x}=\frac{VLV^{T}a-WRW^{T}b}{VLV^{T}-WRW^{T}}.$$ (7)

Once the transition state is identified, the difference in energy between the two equilibrium states can then be evaluated as

$$\Delta E=\frac{{\left(\bar{x}-b\right)}^{T}P\left(\bar{x}-b\right)}{2}-\frac{{\left(\bar{x}-a\right)}^{T}Q\left(\bar{x}-a\right)}{2},$$ (8)

Δ*E* can also be defined as the energy difference between the transition state energies relative to the two equilibrium states.

In Equations (6) and (7), the unknowns are $\bar{t}$ and $\bar{t}-t_{f}$, which can also be written as ${\bar{t}}_{l}$ and ${\bar{t}}_{r}$, such that ${\bar{t}}_{l}+{\bar{t}}_{r}=t_{f}$. As noted earlier, estimating the input *t_f_* is crucial for generating a correct transition state using PATH. As the parameters Δ*E* and *t_f_* are related via the convergence surface (Fig. 2), this also means that evaluation of *t_f_* is essential for the estimation of Δ*E*.

Simulations of several systems using PATH indicate that the structure of the transition state becomes invariant as *t_f_* is large. In the case of the 1D diatomic system, we generated the convergence surface and calculated the Δ*E* values for different $\bar{t}$ and *t_f_* values. For a constant $\frac{\bar{t}}{t_{f}}$ of 0.5, we observed that at large values of *t_f_* the values of Δ*E* are invariant. This also means that the structure of the transition state is a constant. This result implies that the value of *t_f_* is immaterial as long as it is large but this assumption gives rise to another problem with PATH parameters. At extremely large values of *t_f_*, we observe that the path spends most of its time near the equilibrium structures and uses a fraction of the total time to change the conformation of the protein. Also we observed that the system spent more time in the narrower (more energetic) well than in the wider well. This behavior contradicts statistical mechanics. But, at the same time, once the conformational change starts, the system takes less time to climb up the potential well in the narrower well than in the wider well, which is consistent with statistical mechanics.

The origin of these behaviors can be understood in the following way. As described in more detail in Appendix A, converting the equations of motion from those defined by classical action to those defined by OM action changes the fractional increment in position, *x*(*t*), from an oscillatory motion to the hyperbolic sine function in (1). As a consequence, the system invariably spends most of its time at the origin (i.e., at *x*(*t*) = *a*) and commences its climb to the transition state after an inordinately long time. This problem of the system spending most of the time in the initial state has previously been observed.^17,19,33^ As was true of the analytical gradient provided in the string method, a solution to this problem can be obtained by transforming the Lagrangian from the time-dependent Newtonian description to the dual, energy-dependent Hamilton-Jacobi description.^16^ That elegant coordinate transformation affords a more complete solution to the problem. It is possible that for complex dynamic processes like *ab initio* protein folding, where important structural changes may occur at the level of bond vibration, neglecting part of the trajectory may entail the loss of relevant information.

For protein conformational changes, like domain motions that depend on large frequency rigid-body motions, we describe multiple lines of evidence that no essential information is lost by truncating the initial, invariant portion of the trajectory during which the structure does not change. To resolve this problem, we realized that the system must be given just enough time for the transition state to converge and no more. We therefore truncate the PATH trajectory by beginning only when the system has moved away from *a* by at least 10% of the total distance between the equilibrium state and the transition state. This is an arbitrary choice; using 1% of the distance from *a* would change the resulting transition state almost imperceptibly.

An appropriate value of *t_f_* can be calculated for the 1D diatomic system in the following way. Using (1), a general trajectory equation can be written as

$$x\left(t\right)=a+\left(\bar{x}-a\right)\frac{\sinh\left(kt\right)}{\sinh\left(k\bar{t}\right)},$$ (9)

when $\bar{t}\to \infty$, (9) becomes

$$x\left(t\right)=a+\left(\bar{x}-a\right)e^{-k\left(\bar{t}-t\right)}.$$ (10)

As we are interested in the time at which the system has changed by at least 10%,

$$e^{-k\left(\bar{t}-t\right)}=0.1,$$ (11)

which gives

$${\bar{t}}^{opt}\Rightarrow \bar{t}-t=\frac{2.302}{k}.$$ (12)

This equation directly computes $\bar{t}$ for a 1D diatomic system but for multiatom systems in 3D, there are multiple interatomic interactions, and hence multiple force constants associated with the diagonalized Hessian matrix. Hence, we calculated the average force constant for a structure which is the average of the trace of the Hessian

$$\bar{k}=\frac{tr\left(H\right)}{3N},$$ (13)

where *N* is the number of atoms.

### The new path algorithm avoids an iterative search

The MinActionPath algorithm^18^ calculates the structure of the transition state $\bar{x}$ using the velocity continuity equation (6) by assuming a value of $\bar{t}$ based on the given value of *t_f_*. Then, this $\bar{x}$ is validated by checking if it satisfies the energy equation (8) for a given value of Δ*E*. If $\bar{x}$ does not satisfy the energy equation, then a new value of $\bar{t}$ is assumed and a new $\bar{x}$ is identified. This process is repeated until a value of $\bar{t}$ is found for which $\bar{x}$ satisfies the energy continuity requirement.

In the new algorithm, using (13), ${\bar{t}}^{opt}$ is directly evaluated from the force constants. This ${\bar{t}}^{opt}$ is used to identify the transition state structure directly, without iteration, which speeds up the PATH calculations by an order of magnitude, thereby simplifying, and substantially increasing the speed of an already fast method. Using this modified algorithm to calculate ${\bar{t}}^{opt}$, we calculated the transition state for three different systems and compared the structures with those generated from other simulation methods.

## RESULTS

First, we show that although PATH and two other computational approaches produce different low-energy structures connecting the ground-states with the transition state, all three methods agree closely on the configurations of their transition states. Second, we show that the PATH transition states are close to the saddle points of free-energy surfaces connecting initial and final states generated by replica-exchange Discrete Molecular Dynamics simulations.^10,11,34^ We show that aromatic side-chain rearrangements create similar potential energy barriers in the transition-state structures identified by PATH for a signaling protein, a contractile protein, and an enzyme.

### PATH and ANMPathway trajectories agree most closely with string method trajectories at their transition states

**FIG. 4.**
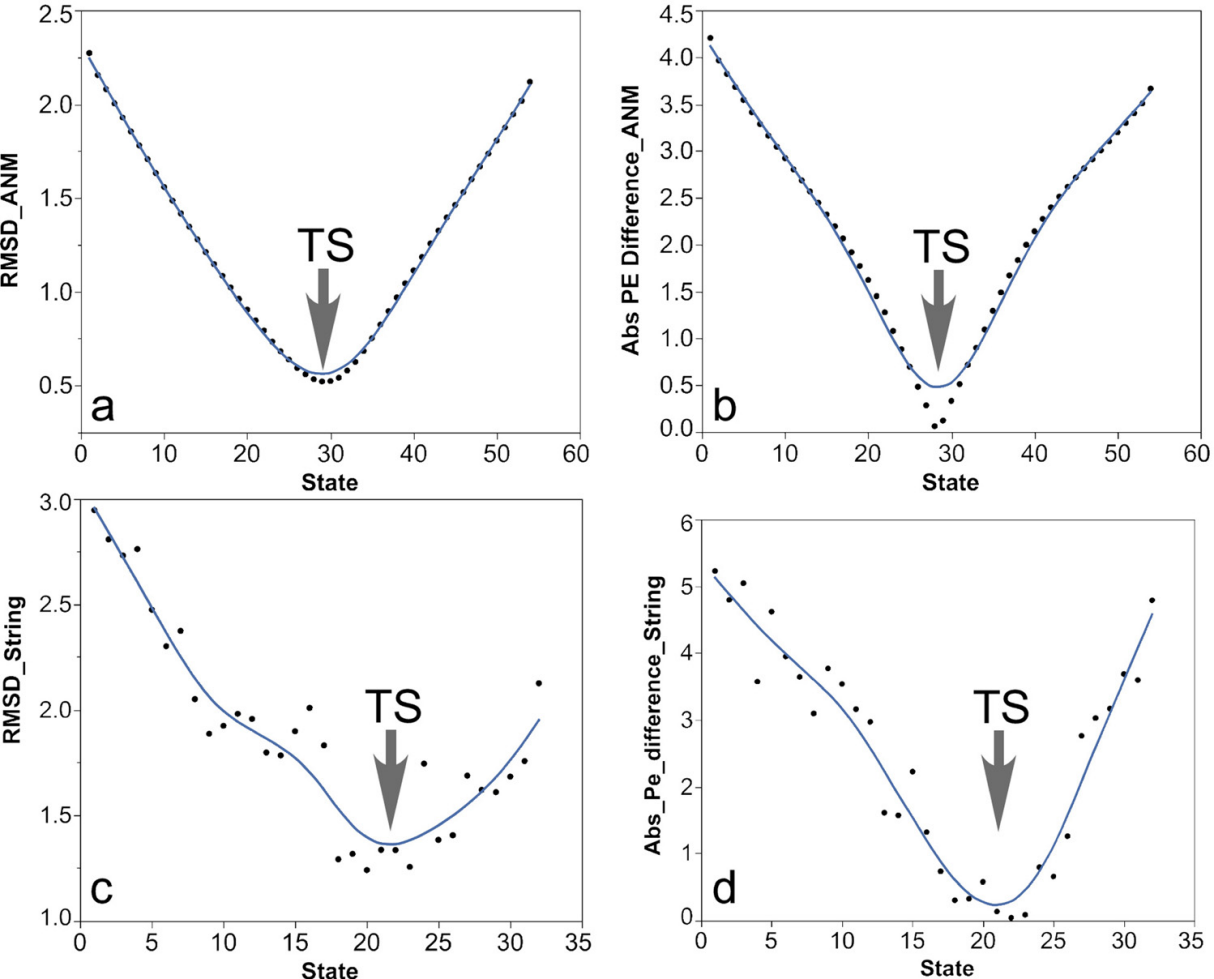
The ANMPathway trajectory and the string trajectories were compared with the PATH trajectory. In (a), we calculated the RMSD between the transition state from the PATH trajectory and all the states along the ANMPathway trajectory. States 28–31 are structurally similar to the PATH transition state. In (b), we calculated the potential energy (PE) of each state in the ANMPathway trajectory with respect to the potential energy well of the initial and the final state and their absolute difference was plotted. States 27–30 have the lowest potential energy difference, which coincides with the states in (a). We performed a similar comparison between the string trajectory (G3c) and the PATH transition states in (c) and (d). States 18–23 are structurally similar to PATH transition state, and the same states also have the lowest potential energy difference, implying their proximity to the same transition state.

We compared trajectories from the simulations of the converter domain from myosin VI performed using the string method,^31^ ANMPathway, and PATH. Since the reaction coordinates of the three trajectories are different, it would be difficult to compare them at every instant. We compare in Fig. 4 the structural similarity and energetic properties of the string transition state as evaluated according to the linearized ANM force field used by PATH. For both comparisons, subset of structures in the string trajectory that was structurally most similar to the PATH transition state (Fig. 4(a)) was the same subset for which the absolute potential energy difference between those calculated with respect to the initial and final states, was closest to zero (Fig. 4(b)). In the context of PATH, the structure whose corresponding potential energy difference is zero is, by definition, the transition state.

We performed a similar analysis with the myosin conformational change trajectory from the ANMPathway method.^8^ We found that when the PATH transition state is compared with the ANMPathway trajectory, the structures are the closest [root mean squared deviation (RMSD) 0.52 Å] near the transition state of the ANMPathway trajectory [Fig. 4(c)]. Similarly, the same group of structures have the absolute potential energy difference closest to zero [Fig. 4(d)].

### Discrete molecular dynamics replica exchange simulations verify that transition states identified by path are close to saddle points in the free energy surface connecting initial and final states

The main reason we undertook to study conformational transition state structures was to extend what previously had been established for the structural reaction profile of the *B. stearothermophilus* tryptophanyl-tRNA synthetase (TrpRS; Kapustina *et al*.^21^). TrpRS passes through three distinct structural states:

• an Open state that can be stabilized either by stoichiometric amounts of tryptophan or by sub-stoichiometric amounts of Mg·ATP (adenosine triphosphate)
• a closed, Pre-TS, stabilized by stoichiometric amounts of Mg·ATP and a tryptophan analog
• a closed, Product state (Pdt), stabilized either by the bound intermediate adenylate product, tryptophanyl-5′AMP, or by stable analogs thereof

As the ligands bind to the Open state, the protein undergoes an induced fit conformational change and goes to the Pre-TS state. At the Pre-TS state, a subsequent catalytic step takes the Pre-TS state to the Pdt state. Both induced-fit and catalysis are slow, relative to the chemical transformation of the substrates; each is therefore associated with a different conformational transition state. Preliminary analysis with the PATH program had given us descriptive accounts of the two transitions.

• Induced-fit proceeds by an early and higher energy barrier that matches the behavior seen by MD simulations of the TrpRS monomer^23^
• Catalysis proceeds by a later, lower barrier transition state in which the volume of the tryptophan binding pocket assumes a minimum value immediately after the conformational transition state identified by PATH.^41^

The earlier MD calculations relating to the Induced-fit transition were short, 10 ns simulations, and represented what appeared to be a slower conformational change. As MD simulations led to a confirmation of the results PATH had given for the Induced-Fit transition,^23^ we decided to see whether similar, but more detailed simulations might allow a more stringent test of results the PATH algorithm had given for the catalytic transition. As the catalytic transition represents what is likely a more rapid conformational change with a lower barrier, we carried out replica exchange calculations using DMD,^10,11,34^ with sufficiently long equilibrations to appropriately sample the free energy surface connecting the Pre-TS and Pdt states.

DMD simulations were set up with the same configuration of ligands that we had used for PATH: AMP (adenosine monophosphate), Tryptophan, and Pyrophosphate. These ligands were configured as before^41^ to allow an approximation to the actual chemical reaction displacing pyrophosphate from ATP with tryptophan. From the resulting snapshots, we computed the internal coordinates used previously to describe the Induced-Fit transition (Twist and Hinge^20^). Sufficiently many steps were computed to visualize the relative populations centered on the two states. For each case, we identified representative structures for the two different distributions. Free energy surfaces were then computed by fitting a bi-variate quadratic to the points between the two equilibrium states. These representative structures reflect the stable, equilibrium structures of the two states in the DMD force field^9,34^ as obtained from the DMD simulations. They were then input as initial and final states to PATH.

**FIG. 5.**
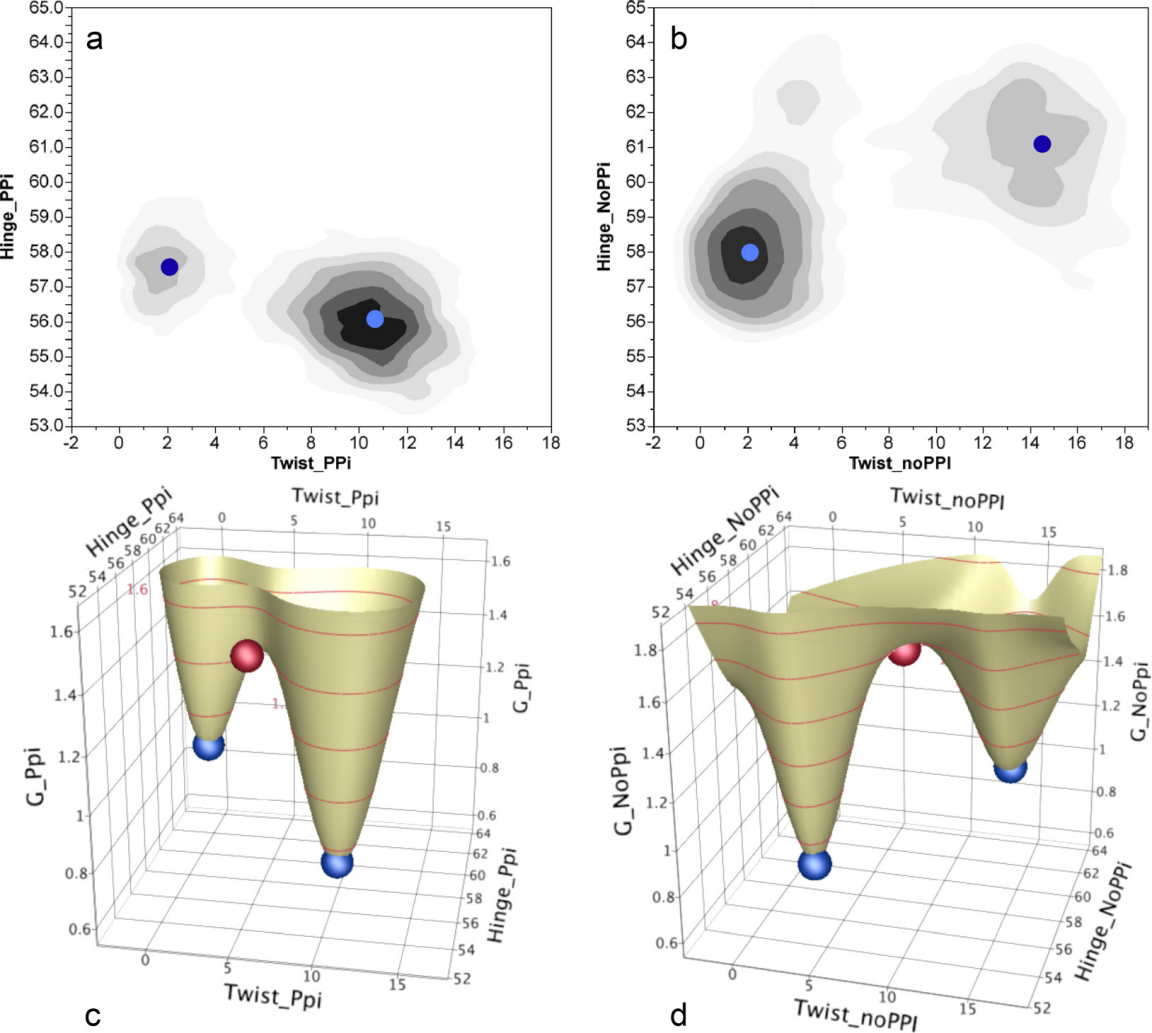
Free energy surfaces for the fully liganded TrpRS monomer derived from DMD replica exchange computations and plotted as a function of the two conformational angles, Twist and Hinge, which represent collective variables for the catalytic conformational change derived by Kapustina.^20^ The structures (2000 snapshots) generated at the lowest DMD temperature (∼175 K) were used in the analysis. (a) Distributions of the TrpRS Pre-transition state and Product derived from simulations initiated from the Product state in the (harmonically restrained) presence of AMP, tryptophan, and pyrophosphate. (b) Distributions of these two states in similar simulations without pyrophosphate and without restraining potentials. In (a) and (b), the dark blue circles represent the free energy minima of the less populated state fitted to a bivariate quadratic response surface. Light blue circles represent free energy minima computed using the same approach for the more highly populated states. (c) Free energy surface derived from (a). (d) A similar plot derived from (b). Blue spheres represent the initial and final states input to PATH computations; red spheres represent the coordinates of the transition states produced by PATH.

These calculations produced two notable results:

• The apparent free energy difference between the Pre-TS and Product states depends strongly on the presence of the bound product, pyrophosphate (PPi). If the PPi was retained in the binding pocket by a harmonic potential, the equilibrium was far on the side of the Pre-TS state (Fig. 5(a)). On the other hand, if this potential (or constraints) mimicking PPi binding was relaxed or omitted, we observe rapid PPi release and the distribution of states exhibits higher probability towards product state (Fig. 5(b)). This behavior is especially interesting in view of the possibility that early release of orthophosphate following actin binding triggers the myosin V powerstroke.^32^
• Transition states for the transitions with and without the harmonic potential restraining the PPi output by PATH fall close to the coordinates of the saddle points of the respective energy surfaces (Figs. 5(c) and 5(d)).

### Transition states identified by PATH display comparable rate-limiting structures in three different systems

**FIG. 6.**
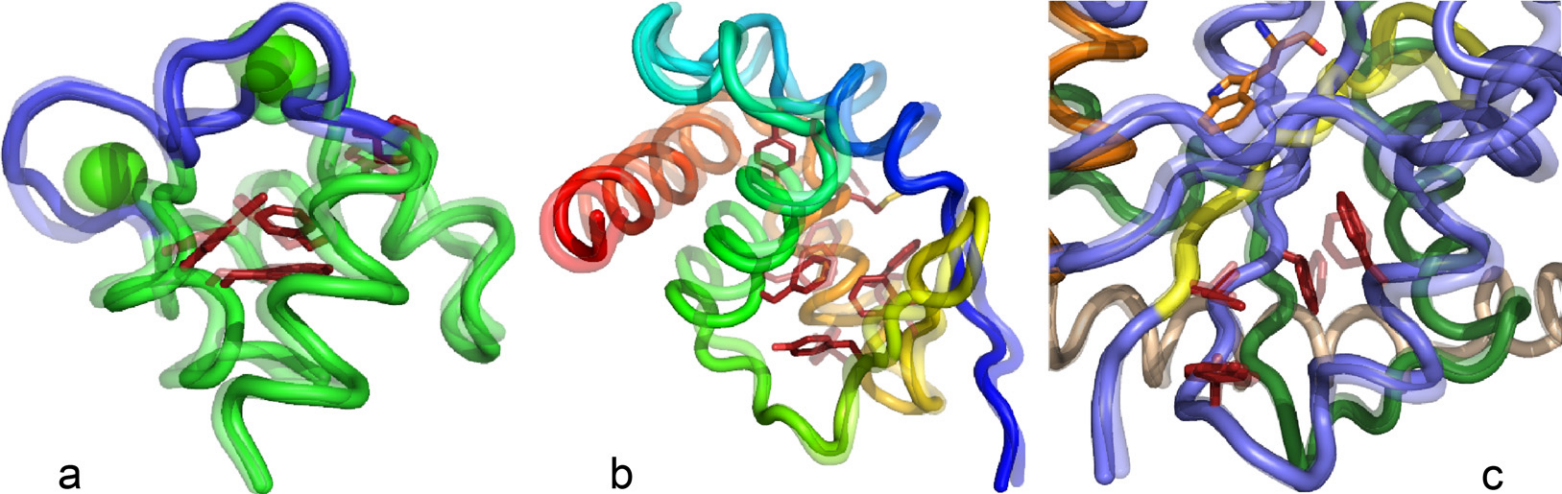
Conformational transition state structures for Calmodulin Ca^2+^-binding domain (a), Myosin VI converter domain rigor to Prepowerstroke (b), and the TrpRS induced-fit (c) transition states. Aromatic residues that flip at the transition state are highlighted in red. The initial state is 50% transparent, to distinguish the states before and after the rate-limiting step.

We began these studies to access structural information about the transient conformational transition state(s) that appear to be rate-limiting for TrpRS catalysis.^21^ In the course of the work, we found it useful also to investigate PATH behaviors of other model systems, including the 1D system described earlier here. Two well-defined protein conformational transitions–Ca^2+^release by the Ca^2+^-binding domain of calmodulin and the converter domain of myosin VI–also proved useful in verifying the generality of aspects described in the Theory of PATH section. These studies reveal a remarkable similarity in all three transition-states (Fig. 6). In each case, the rate-limiting conformational change involves re-packing of multiple aromatic side chains^4,5,22^ associated with subtle rearrangements of the surrounding backbone chains. Such rearrangements are known to occur on a far slower timescale (*μ*s to ms (Ref. 35)) than rotamer exchanges of aliphatic side chains in hydrophobic core regions. Further, the timings of the three transition states (middle, late, early) are consistent with the overall equilibrium constant for the conformational change, via Hammond's postulate.

## CONCLUSIONS

We have reviewed a substantial literature on the methodology of computing trajectories for conformational changes from what is essentially a hybrid between technical and lay perspectives, consistent with our own interest in the biochemical importance of conformational transition states. In the process, we have de-mystified much previous work in what we hope are useful ways. A remarkable aspect of that literature is that it has yet to describe either the structural details of the cooperative side chain behavior that comprise the barrier or the functional implications of conformational transition states. To contribute usefully to that discussion, we have here tried to address these two aspects of conformational trajectories.

PATH affords a rapid, accurate way to assess the structural features that limit the rate of conformational changes. PATH computations can be performed in a manner that circumvents the problem of defining the four required input parameters. In identifying the mathematical origin of the time-course anomalies and a workable algorithm for choosing appropriate *t_f_* values, we have established a basis for more widespread use of the PATH algorithm. One important potential benefit of the broader investigation into details of conformational transition states would be to facilitate the identification of specific residues likely to be involved in allosteric communication, as we have done with TrpRS,^21,40,41^ thereby enhancing the role of combinatorial mutagenesis to investigate higher-order coupling in protein functions.

We have provided persuasive evidence that minimization of the Onsager-Machlup action with the PATH algorithm produces a transition state in good agreement with that provided by the String Method and by ANMPathway. The most probable path is generally considered to define a smooth curve through the center of a tube in pathspace.^14^ Opinions differ, however, over the effective diameter of such a tube, and/or whether multiple tubes might pass through different transition states.^26^ We show here that three distinct algorithms based on different force fields, using different sets of collective variables to define the pathspace identify quite similar transition state structures. That observation suggests that functionally distinct domain configurations in proteins are separated by well-defined structural barriers.

Further, transition states identified by PATH coincide closely with stationary points of free energy surfaces derived using replica-exchange DMD simulations. That evidence validates the use of PATH by a wider group of potential users interested in structural details of the cooperative side chain rearrangements^26^ that limit conformational changes in proteins of interest. Further, our demonstration that residues involved in limiting the TrpRS conformational changes can be implicated in long-range coupling to the active site^40,41^ suggests that PATH may be useful in identifying candidates for a broad range of combinatorial mutational analyses of enzymatic Fig. 6(c), signaling Fig. 6(a), and contractile Fig. 6(b) mechanisms.

The observation that quite similar side-chain configurations limit the conformational changes of three quite distinct proteins points to a more general phenomenon in which nature chooses to build multi-state behavior in similar ways. This conclusion has potentially deeper significance because the transition states we have described are more or less independent of whether side chains are included in the simulations. ANMPathway simulations are necessarily performed with only *Cα* atoms, and we performed PATH simulations both with all-(heavy)atom and *Cα* only coordinate files. The resulting transition states are almost indistinguishable (RMSD = 0.25 Å for *Cα* atoms). We cannot account for this coincidental behavior except to note that it suggests a higher-order coupling between side-chain and backbone behavior.^7,24^ That behavior is, however, reminiscent of our observation that combinatorial point mutation of residues limiting the rate of domain movement during induced-fit in TrpRS show that those residues are coupled to the catalytic activation of the active-site Mg^2+^ ion^40^ and to specific recognition of tryptophan^41^ by essentially the same free energies as those coupling the anticodon-binding and the CP1 insertion domains to catalysis and recognition.^25^

## MATERIALS AND METHODS

### Structures

We use three TrpRS structures in our studies. These structures were derived from the crystal structures of the three conformations of TrpRS, namely, Open (1MAW,1MB2), Pre-TS (1MAU) and Pdt (1I6L). We excised the terminal aminoacid (R328) from the structures as it is not observed in most of the crystal structures. We believe that its absence would not affect the conformational change of the rest of the protein. The ligands in the binding pockets are different for the different states of TrpRS. To make the ligands consistent in all the three structures, we used Tryptophan, AMP, PPi as separate molecules in the binding pocket; the distance between these molecules changes, depending on the state and the chemical species that they represent. We have previously used a similar arrangement^23^ and this allows approximating the chemical reaction without requiring the use of quantum calculations. The myosin VI structures for the rigor state and the prepowerstroke state were derived from 2BKH and 2V26, respectively. As described in Ref. 31, only residues 703–788, which form the converter domain, were used in the simulations. The equilibrium structures for calmodulin were derived from 1CMF and 1FW4.

### Path simulations

To run PATH simulations, the number of atoms in the two equilibrium states and their relative order in the two pdb files must be the same. Only the heavy atoms are used. The modified algorithm requires no input parameters other than the two equilibrium states, because the force constants are assumed to be 0.01 for both states, and errors in this assumption are compensated by the evaluation of $\bar{t}$ for the forward and reverse reactions from Eq. (12). For purposes of comparison, we note that the free energy surfaces (Fig. 5) from which we estimated the transition state structure using replica exchange DMD simulations took 10^4^ times longer than the PATH calculation. The new PATH algorithm is available from the author.

### ANMPathway simulations

The ANMPathway calculations^8^ were set up on the ANMPathway server. Default input force constants = 0.1 were used for both the energy wells. All the other parameters were set to their default values–Cutoff - 15 Å, Energy offset - 0, Step size (on cusp) - 0.8, Step size (slide down) - 0.04 and Target RMSD - 0.1 Å.

### DMD simulations

Replica Exchange Discrete Molecular Dynamics (REX/DMD) simulations were set up with the Pdt state structure described previously. A harmonic potential was applied between the atoms of the ligands and all the surrounding atoms within 3.5 Å to retain the ligands within the binding pocket. In general, replica exchange simulations are used for efficient sampling of the conformational landscape of a given system. However, we were only interested in monitoring the transition between the Pdt and Pre-TS state. To facilitate the exploration of this particular transition event as well as to expedite the sampling, we introduced weak harmonic constraints to guide the system progressing from Pdt to Pre-TS state. By comparing the native contacts within the two systems (as obtained from their crystal structures), we extracted the unique contacts that were present in the Pre-TS and not the Pdt state. Those contacts were used as experimental constraints. The DMD force field is currently equipped to work only with Cu^2+^ or Zn^2+^. Since ATP is complexed with Mg^2+^in in the Pre-TS state, we replaced it with Zn^2+^. We believe that this replacement would not affect the conformational change of the protein in a significant way. We simulated parallel replicas at 24 temperatures ranging from ∼175 K to ∼405 K for a total duration of 2.5 million steps (∼125 ns) as described in detail in Ref. 42. As the system requires 500 000 steps to equilibrate, all our analyses were performed with the remaining 2 million steps. Snapshots were generated every 1000 steps, hence all our analyses (Fig. 5) include 2000 snapshots.

### Fitting the free energy surfaces

Each of the 2000 snapshots from the lowest temperature replica exchange DMD simulation was segregated in 225 bins of equal size, based on their Hinge and Twist angles. Based on the distribution of structures within these bins, the free energy surface is computed using the formula

$$\Delta G=-k_{B}Tln\left(100*\left[\frac{n_{i}}{N}\right]\right),$$

where *n_i_* is the number of structures in the i*th* bin and N is the total number of structures.

Then, these free energy values are fitted to the following equation to generate the free energy surfaces in Fig. 5,

$$\begin{array}{c} \Delta G=C+Ae^{-\left(\frac{{\left(X-Tw1\right)}^{2}}{2SigTw1}+\frac{{\left(Y-H1\right)}^{2}}{2SigH1}+\frac{J\left(X-Tw1\right)\left(Y-H1\right)}{2\sqrt{SigTw1^{2}+SigH1^{2}}}\right)}+Be^{-\left(\frac{{\left(X-Tw2\right)}^{2}}{2SigTw2}+\frac{{\left(Y-H2\right)}^{2}}{2SigH2}+\frac{L\left(X-Tw2\right)\left(Y-H2\right)}{2\sqrt{SigTw2^{2}+SigH2^{2}}}\right)}\\ +D\left(X-Twt\right)+F{\left(X-Twt\right)}^{2}+G\left(Y-Ht\right)+H{\left(Y-Ht\right)}^{2}, \end{array}$$

where X and Y are the Twist and Hinge angles and the constants Tw1, H1, Tw2, and H2 are the twist and hinge, respectively, of the Pdt and Pre-TS structures and Twt and Ht are coordinates of the saddle point.

## APPENDIX A: EQUATIONS OF MOTION FOR THE MOST PROBABLE PATH

PATH is based on the ANM, in which the interatomic interactions are modeled on vibrating springs. In general, such a system follows Newtonian mechanics and the path that the system takes is the path of least action. In addition to that, PATH considers the dynamics of such a system to be stochastic in nature and models the system to follow the Langevin equation of motion and replaces the classical action with the OM action. In this section, we describe PATH and OM action by comparing them with the familiar classical spring system.

### Classical action

Consider a 1D diatomic system following Newtonian dynamics in a single potential well. The equation of motion can be derived by identifying the path that minimizes action. Since the path is deterministic, the probability, *p*, of this path is always equal to 1. But to compute this path, it is required to know the kinetic and potential energies of the system.

Potential energy can be calculated from the interaction between the two atoms. As mentioned previously, the two atoms are considered to be connected by a Hookean spring, and the interaction between the atoms is considered to be harmonic in nature. Hence, we write the potential energy as

$$V\left(x\right)=\frac{k}{2}{\left(x-a\right)}^{2},$$ (A1)

where *a* is the equilibrium distance between the two atoms, and *x* − *a* is the displacement from the equilibrium position *a*.

Since the kinetic energy of the system is $T=\frac{1}{2}m{\dot{x}}^{2}$, we write the Lagrangian as

$$L=T-V=\frac{1}{2}\left(m{\dot{x}}^{2}-k{\left(x-a\right)}^{2}\right).$$ (A2)

Using (A2), we write the equation for classical action as

$$S_{cl}=\int_{0}^{t}L.dt=\int_{0}^{t}\frac{1}{2}\left(m{\dot{x}}^{2}-k{\left(x-a\right)}^{2}\right)dt.$$ (A3)

Equation (A3) computes the action of any given path but we have to find the path of minimum action. Since action is a functional, its extremum is calculated using a variational principle. In Lagrangian mechanics (using the Lagrangian to derive Newton's equation of motion), this boils down to finding the solution to the Euler-Lagrange equation

$$\frac{\partial L}{\partial x}-\frac{d}{dt}\left(\frac{\partial L}{\partial \dot{x}}\right)=0.$$ (A4)

On applying the boundary conditions, *B*_1_ and *B*_2_, which are, at time *t*_1_, *x*(*t*_1_) = *x*_1_ and *t*_2_, *x*(*t*_2_) = *x*_2_, the solution to the Euler-Lagrange equation gives the following trajectory equation:

$$x\left(t\right)=a+\frac{1}{\;\text{sin}\left(\omega \left(t_{2}-t_{1}\right)\right)}\left[\left(x_{1}-a\right)\text{sin}\left(\omega \left(t_{2}-t\right)\right)-\left(x_{2}-a\right)\text{sin}\left(\omega \left(t_{1}-t\right)\right)\right],$$ (A5)

where $\omega =\sqrt{\frac{k}{m}}$ is the angular frequency. This is the equation of motion of a spring following Newtonian dynamics which also minimizes classical action with boundary conditions *B*_1_ and *B*_2_.

To calculate the action from (A3), we calculate the velocity of the system, which is

$$\dot{x}\left(t\right)=\frac{1}{\;\text{sin}\left(\omega \left(t_{2}-t_{1}\right)\right)}\left[-\omega \left(x_{1}-a\right)\text{cos}\left(\omega \left(t_{2}-t\right)\right)+\omega \left(x_{2}-a\right)\text{cos}\left(\omega \left(t_{1}-t\right)\right)\right],$$ (A6)

and the total action of this path is

$$S_{cl}=\frac{m\omega }{2\;\text{sin}\left(\omega \left(t_{2}-t_{1}\right)\right)}\left[\left[{\left(x_{1}-a\right)}^{2}+{\left(x_{2}-a\right)}^{2}\right]\text{cos}\left(\omega \left(t_{2}-t_{1}\right)\right)-2\left(x_{1}-a\right)\left(x_{2}-a\right)\right].$$ (A7)

### Protein conformational change is a stochastic process

Unlike the classical spring, dynamics of protein molecules cannot be considered to be deterministic but rather a stochastic process, which is modeled by an overdamped Langevin dynamics equation

$$m\gamma \dot{x}=-\frac{dV\left(x\right)}{dx}+\sqrt{2m\gamma k_{B}T}\xi ,$$ (A8)

where *γ* is the diffusion coefficient, and *ξ* is a delta-correlated Gaussian random (zero mean) force. That is

⟨ξ(t)⟩=0, (A9)

⟨ξ(t)ξ(t′)⟩=δ(t−t′). (A10)

In order to understand the Langevin equation, consider the same diatomic 1D system. Unlike the deterministic and periodic equation for a classical spring, if the Langevin equation describes a stochastic process, it can only calculate probabilities of paths that the system might take. Given the current state *x*_1_ at time *t*_1_, the probability of reaching state *x*_2_ at a small time increment Δ*t*, assuming microscopic reversibility, is given by

$$p\left(x_{2}\;\text{at}\;t+\Delta t|x_{1}\;\mathrm{at}\;t\right)=\frac{e^{-\frac{k}{4k_{B}T}{\left(\left(x_{1}-a\right)-\left(x_{2}-a\right)e^{\frac{k\Delta t}{m\gamma }}\right)}^{2}\left(-1+\coth\left(\frac{k\Delta t}{m\gamma }\right)\right)}}{{\left(\frac{1}{4\pi k_{B}T}\left[k\left(1+\coth\left(\frac{k\Delta t}{m\gamma }\right)\right)\right]\right)}^{-\frac{1}{2}}},$$ (A11)

where *k_B_* is the Boltzmann constant, and *T* is the temperature. This is the solution to the Fokker-Planck equation.

If the total path is a succession of such states, then the joint probability can be calculated as the product of the probabilities of the individual segments. But since we are interested only in the probability of the most probable path, we calculate this by minimizing the exponent in (A11). To do this, Onsager and Machlup^29^ developed an interesting method to calculate the trajectory by considering Equation (A11) to be of the form

$$p\propto e^{-\frac{S_{OM}}{2mk_{B}T}}.$$ (A12)

By treating the numerator of the exponent to be analogous to classical action, they were able to simplify it into an integral of the form

$$S_{OM}=\frac{1}{2\gamma }\int_{0}^{t}{\left(m\gamma \dot{x}+k(x-a)\right)}^{2}dt,$$ (A13)

and the functional *S_OM_* is now referred to as the *Onsager-Machlup action*.

Since we have a functional whose minimum value has to be calculated, it can be done by solving the Euler-Lagrange equation and on application of the same boundary conditions as in the classical case, *B*_1_ and *B*_2_, we get the following trajectory equation:

$$x\left(t\right)=a+\frac{1}{\sinh\left(\Gamma \left(t_{2}-t_{1}\right)\right)}\left(\left(x_{1}-a\right)\sinh\left(\Gamma \left(t_{2}-t\right)\right)-\left(x_{2}-a\right)\sinh\left(\Gamma \left(t_{1}-t\right)\right)\right),$$ (A14)

where $\Gamma =\frac{k}{m\gamma }$. This is the equation of motion of the minimum action path undergoing stochastic dynamics, modeled by Langevin equation.

From (A14), we calculate the velocity as

$$\dot{x}\left(t\right)=\frac{1}{\sinh\left(\Gamma \left(t_{2}-t_{1}\right)\right)}\left(\left(-\Gamma \right)\left(x_{1}-a\right)\cosh\left(\Gamma \left(t_{2}-t\right)\right)-\left(-\Gamma \right)\left(x_{2}-a\right)\cosh\left(\Gamma \left(t_{1}-t\right)\right)\right).$$ (A15)

And by substituting (A14) and (A15)) to (A13), we calculate the action as follows:

$$S_{OM}=\frac{mk}{2\sinh\left(\Gamma \left(t_{2}-t_{1}\right)\right)}\left[{\left(x_{2}-a\right)}^{2}e^{\Gamma \left(t_{2}-t_{1}\right)}+{\left(x_{1}-a\right)}^{2}e^{-\Gamma \left(t_{2}-t_{1}\right)}-2\left(x_{2}-a\right)\left(x_{1}-a\right)\right].$$ (A16)

## APPENDIX B: CONSTRUCTING THE HESSIAN FOR A LINEARIZED ANM POTENTIAL

PATH uses a linearized ANM potential to represent interatomic interactions where each atom pair is connected to each other in some manner via springs with a single force constant k. According to ANM,^1^ between any two atoms, then, we have the pair potential

$$U\left(r^{i},r^{j}\right)=\frac{1}{2}k{\left(r-\bar{r}\right)}^{2},$$ (B1)

where *r^i^* is the position of the *i*th atom, *r^j^* the position of the *j*th atom. We form the distance *r* by taking the magnitude of $r\equiv r^{i}-r^{j}$, pointing from the *j*th atom to the *i*th one. This distance is compared to the equilibrium length, the magnitude of $\bar{r}\equiv {\bar{r}}^{i}-{\bar{r}}^{j}$, where the set ${\left\{{\bar{r}}^{i}\right\}}_{i=1}^{N}$ is a specified equilibrium configuration defined by the input crystal structure. This potential still yields a nonlinear set of equations of motion, so we will linearize it and obtain our final effective potential for the system following.

Consider a small displacement from the equilibrium configuration: *δr^i^* and *δr^j^*, then we can construct $\delta r\equiv \delta r^{i}-\delta r^{j}$. The potential, for small *δr* reads

$$U=\frac{1}{2}\frac{s}{{\bar{r}}^{2}}\sum_{p,q}{\bar{r}}_{p}{\bar{r}}_{q}\delta r_{p}\delta r_{q},$$ (B2)

where the subscripts *p* and *q* represent the *x*, *y*, and *z* Cartesian coordinates of the vectors. We can define the Hessian to be the matrix implicit in the above summation

$$h_{ij}\;≐\;\frac{s}{{\bar{r}}_{ij}^{2}}\left(\begin{array}{ccc} {\bar{r}}_{x}^{2} & {\bar{r}}_{x}{\bar{r}}_{y} & {\bar{r}}_{x}{\bar{r}}_{z}\\ {\bar{r}}_{y}{\bar{r}}_{x} & {\bar{r}}_{y}^{2} & {\bar{r}}_{y}{\bar{r}}_{z}\\ {\bar{r}}_{z}{\bar{r}}_{x} & {\bar{r}}_{z}{\bar{r}}_{x} & {\bar{r}}_{z}^{2} \end{array}\right).$$ (B3)

This is the Hessian appropriate to the linearization of the spring connecting atom *i* with atom *j*, but we have many such connections in general. Here, *s* is a scale constant that is generally derived from fitting the mean square fluctuation of the atoms to the crystallographic B values.^2^ For non-high resolution crystal structures and for computational mutants, since the B values cannot be used to estimate the scale constants, we assume 0.01 as the scale constant for both the structures. Because the argument of the sinh term in the OM equation of motion [Eq. (1)] is the product of the force constant and *t_f_*, giving a reciprocal impact of those two parameters [Eq. (12)], we believe that any error induced by this assumption will be compensated by the estimation of *t_f_* from the average force constant $\bar{k}$.

We want to build the full Hessian *H* from these three-by-three blocks. If we refer to the three coordinates of the *i*th atom as *x_i_* in *x* (so that *x* has *N* such entries), and *H_ij_* gives the three-by-three block of *H* at row *i*, column *j*, then the Hessian is constructed by adding *h_ij_* from (B3) to *H_ii_* and *H_jj_* and subtracting *h_ij_* from *H_ij_* and *H_ji_*.

In the end, we have a symmetric matrix *H* ∈ *R*^3*N*×3*N*^, and an equilibrium configuration *a* ∈ *R*^3*N*^, the effective potential of the system can be written as

$$U=\frac{1}{2}{\left(x-a\right)}^{T}H\left(x-a\right).$$ (B4)

## APPENDIX C: ANALYTICAL DESCRIPTION OF THE CONVERGENCE SURFACE

The convergence surface in Fig. 2 shows that different minimum action paths are generated for different input values of *t_f_* and Δ*E*. Not only do we observe that $\bar{t}$ varies with different values of these two input parameters but also we understand that the velocity continuity equation (6) gives rise to this surface.

Though the convergence surface is observed for large proteins and for 1D diatomic systems alike, we can write a simplified equation only for the latter. In this section, we will derive an equation for the convergence surface of the diatomic system from the velocity continuity equation.

For the diatomic system in 1D, the most probable trajectory is calculated separately for the left side and the right side from (A14) as (1) and (2). From those two equations, we can derive the velocity continuity equation as

$$k_{r}\left(\bar{x}-b\right)\coth\left(k_{r}\left(\bar{t}-t_{f}\right)\right)=k_{l}\left(\bar{x}-a\right)\coth\left(k_{l}\bar{t}\right).$$ (C1)

To further simplify this equation, we have to know the structure of the transition state ($\bar{x}$), which, in a 1D diatomic system, can be computed by rearranging Equation (8) to

$$\frac{1}{2}k_{l}{\left(\bar{x}-a\right)}^{2}+\Delta E=\frac{1}{2}k_{r}{\left(\bar{x}-b\right)}^{2}.$$ (C2)

If the initial structure is *a* = (*a*_1_, *a*_2_) and the final structure is *b* = (*b*_1_, *b*_2_), then we can calculate the structure of the transition state $\bar{x}=\left(x_{1},x_{2}\right)$ by writing the energy continuity equation as

$$\frac{1}{2}\left(\bar{x}-a\right)\left(\begin{array}{cc} k_{l} & -k_{l}\\ -k_{l} & k_{l} \end{array}\right){\left(\bar{x}-a\right)}^{T}+\Delta E=\frac{1}{2}\left(\bar{x}-b\right)\left(\begin{array}{cc} k_{r} & -k_{r}\\ -k_{r} & k_{r} \end{array}\right){\left(\bar{x}-b\right)}^{T}.$$ (C3)

The two matrices in Equation (C3) are the Hessian matrices of the initial and final states, as outlined in Appendix B.

We can rewrite Equation (C3) as

$$\begin{array}{c} \frac{k_{l}}{2}\left(\begin{array}{cc} x_{1}-a_{1} & x_{2}-a_{2} \end{array}\right)\left(\begin{array}{cc} 1 & -1\\ -1 & 1 \end{array}\right)\left(\begin{array}{c} x_{1}-a_{1}\\ x_{2}-a_{2} \end{array}\right)+\Delta E\\ =\frac{k_{r}}{2}\left(\begin{array}{cc} x_{1}-b_{1} & x_{2}-b_{2} \end{array}\right)\left(\begin{array}{cc} 1 & -1\\ -1 & 1 \end{array}\right)\left(\begin{array}{c} x_{1}-b_{1}\\ x_{2}-b_{2} \end{array}\right). \end{array}$$

On simplification, the above equation becomes

$$\frac{k_{l}}{2}{\left[\left(x_{1}-x_{2}\right)-\left(a_{1}-a_{2}\right)\right]}^{2}+\Delta E=\frac{k_{r}}{2}{\left[\left(x_{1}-x_{2}\right)-\left(b_{1}-b_{2}\right)\right]}^{2}.$$ (C4)

Substituting $\bar{X}=x_{1}-x_{2},\;A=a_{1}-a_{2}$ and *B* = *b*_1_ − *b*_2_, (C4) becomes

$$\frac{k_{l}}{2}{\left(\bar{X}-A\right)}^{2}+\Delta E=\frac{k_{r}}{2}{\left(\bar{X}-B\right)}^{2}.$$ (C5)

Solving for $\bar{X}$

$$\bar{X}=\frac{\left(k_{l}A-k_{r}B\right)-\left(A-B\right)\sqrt{k_{r}k_{l}+\frac{2\Delta E\left(k_{r}-k_{l}\right)}{{\left(B-A\right)}^{2}}}}{\left(k_{l}-k_{r}\right)}.$$ (C6)

For a diatomic system centered on the origin, *x*_1_ + *x*_2_ = 0, giving, together with $\bar{X}$, the transition state, $\bar{x}$.

This transition state structure can be substituted into the velocity continuity equation (C1), and simplified to

$$\frac{\sinh\left(\lambda_{r}t_{f}-\left(\lambda_{r}+\lambda_{l}\right)\bar{t}\right)}{\sinh\left(\lambda_{r}t_{f}-\left(\lambda_{r}-\lambda_{l}\right)\bar{t}\right)}=\left(\frac{\lambda_{r}+\lambda_{l}}{\lambda_{r}-\lambda_{l}}\right)-\left(\frac{2\lambda_{r}\lambda_{l}}{Z_{\Delta E}\left(\lambda_{r}-\lambda_{l}\right)}\right),$$ (C7)

where $Z=\sqrt{\lambda_{r}\lambda_{l}+\frac{2\Delta E\left(\lambda_{r}-\lambda_{l}\right)}{{\left(B-A\right)}^{2}}}$, and *λ_l_* and *λ_r_* correspond to eigenvalues of the respective Hessian matrices.

In any spring system in one dimension, the overall motion is comprised of *N* independent modes, each with its own force constant. In the case of the diatomic system, there is one translational mode, whose force constant is zero and one vibrational mode. Since each mode behaves independently from the other, the spring constant associated with each mode is calculated from the eigenvalues of the respective Hessian matrices. Since *t_f_* is known, we can calculate $\bar{t}$ of the 1D diatomic system, numerically. Thus, the entire landscape of path trajectories shown in Fig. 2 can be computed from Equation (C7). This equation also describes the bi-sigmoidal behavior of the convergence surface. For constant values of *t_f_*, $\bar{t}$ has a sigmoidal relationship to Δ*E*. Similarly at constant Δ*E*, $\bar{t}$ has a sigmoidal relationship to *t_f_*, though the shape of the curve in Fig. 2 is that of a rectangular hyperbola. This behavior rises from the use of positive values of *t_f_*, as negative values of *t_f_* are meaningless.
